# Editorial: Applying cognitive and social psychology to the legal system: what we know today and what is next

**DOI:** 10.3389/fcogn.2025.1695413

**Published:** 2025-10-08

**Authors:** Jeanine Lee McHugh Skorinko, Kimberly Schweitzer, Andre Kehn

**Affiliations:** ^1^Psychological and Cognitive Science Program, Worcester Polytechnic Institute, Worcester, MA, United States; ^2^Department of Psychology, University of North Dakota, Grand Forks, ND, United States

**Keywords:** cognition, psychology and law, social psychology, decision making, bias, police

In memory of Dr. Jeremy Blumenthal (J.D./Ph.D), who was an avid scholar of the intersection between social-cognitive psychology and law, this Research Topic examined the applications of cognitive and social psychology to the legal system today and sought to provide avenues for future empirical work. This Research Topic offers new perspectives on issues and future research related to policing and decision-making in the legal system as well as the influence of sleep, cognitive heuristics, self-affirmation, race, socioeconomic status, victim impact statements, and being a juvenile in the legal system.

## What we know today

Based on the articles submitted, we have learned more about the police and factors that influence decision-making in the legal arena for jurors, judges, and juveniles.

## Police

Mendoza and Caleo offer a thought-provoking piece on why simply hiring more diverse individuals into a police organization will not by itself mitigate racial tensions, and they provide empirically based ideas for increasing diversity in the police to help reduce racial tensions. Relatedly, Smith et al. examine factors that influence support of defunding police initiatives and find that, when the initiative is framed as redirecting funds, it led to more support than framing the initiative as eliminating the police.

## Decision-making

### Race and Socioeconomic Status (SES)

Gilbert et al. provide insight into the complex interactions between juvenile SES and race when assigning judgments of guilt and blame, which at times are mediated by stereotypical judgments. Similarly, Yamamoto and Maeder found that individuals who rely more on heuristic thinking were more likely to perceive biracial targets on trial for a first-degree murder to be less likely to have European ancestry than participants who relied less on heuristic thinking, especially when the defense attorney drew attention to the defendant's race. In addition, Burd et al. examined possible mechanisms to lower the racial disparities in police shootings. Their original research found that when participants were asked to think and write about either self-affirming values or their purpose in life, they were less likely to shoot unarmed targets in a virtual shooting-decision video game. Overall, these studies show that biases, especially those related to race, SES, etc., influence decisions made in the legal arena, and future work needs to continue to investigate these biases and their effects.

### Sleep

Krizan and Curran bring attention to two understudied factors in legal decision-making: sleep and fatigue. They address the roles sleep (or lack thereof) and fatigue may have on those involved in the legal system (e.g., witnesses, victims, confessions) and suggest avenues for future research in this area.

### Judges

Judges play an important role in the decisions made in legal cases. Malegiannaki et al. developed and tested the Judicial Heuristics Assessment Questionnaire (J-HAQ) to better understand how judges utilize different cognitive heuristics in their decision-making process and how this interacts with demographic characteristics of judges. This scale can be a very useful tool for future work.

### Juveniles

Wilford and Frazier provide a thoughtful policy brief on how the legal system should support juvenile suspects, especially when needing to make legal decisions (e.g., take a plea), given the vulnerable state that juveniles are in regarding development, especially neurological development.

### Victim impact statements

Dr. Blumenthal, prior to his passing, examined the role that victim impact statements had on juror emotions and decision-making. Skorinko et al. showcase a collaboration with Dr. Blumenthal before (and after) his passing, examining the role of perspective taking and what the impact of the crime on the victims (high or low) had on juror decision-making. The results from this work showed that victim impact statements influenced perceptions of the defendant, but perspective taking had more limited effects—including with the defendant.

## What is next?

Although exploring different components of the criminal legal system, the research presented in this topic had a common theme: biases that influence the legal arena, from police force to decisions made by those in in the system (i.e., judges, jurors, juveniles). It was also clear from the work submitted that there is still much to learn and more work is needed. This is particularly true for the intersection of race, socioeconomic status, age, and their impact on decisions made within the legal system. Many articles in this Research Topic noted negative outcomes for individuals from historically disadvantaged groups, but we have yet to see empirically based solutions for these injustices. Some articles in this issue, along with social movements, like defund the police and Black Lives Matter, have suggested or attempted reforms, but psycho-legal researchers have yet to empirically test these efforts. For example, Mendoza and Caleo offer four approaches police agencies can take to increase diversity within law enforcement. Future research should test whether these approaches do, in fact, increase diversity and the downstream consequences of having a more diverse police force. Could diversifying law enforcement decrease support for defunding the police (see Smith et al.) and reduce shootings of unarmed citizens (see Burd et al.)? Wilford and Frazier and Gilbert et al. have noted issues with the treatment of juveniles in the legal system; however, more research is needed to examine how these issues can be mitigated. Can automatically providing a lawyer for every juvenile who has contact with the legal system increase fair treatment of juveniles and does this intersect with their identities (e.g., race, gender)? If we decrease biases prevalent in pre-trial procedures, can this help reduce biases researchers have found within the trial procedures with jurors, judges, and witnesses (see Gilbert et al.; Krizan and Curran; Malegiannaki et al.; Skorinko et al.; Yamamoto and Maeder)? If so, may this improve community sentiment regarding the legal system and its fairness? The research described in this Research Topic presents numerous avenues for future research, and we encourage researchers to focus their efforts on developing practical, empirically grounded suggestions to reduce bias in the criminal legal system.

